# Text to Move: A Randomized Controlled Trial of a Text-Messaging Program to Improve Physical Activity Behaviors in Patients With Type 2 Diabetes Mellitus

**DOI:** 10.2196/jmir.6439

**Published:** 2016-11-18

**Authors:** Stephen Agboola, Kamal Jethwani, Lenny Lopez, Meghan Searl, Sandra O’Keefe, Joseph Kvedar

**Affiliations:** ^1^ Partners Connected Health Boston, MA United States; ^2^ Massachusetts General Hospital Boston, MA United States; ^3^ Harvard Medical School Boston, MA United States; ^4^ University of California, San Francisco San Francisco, CA United States; ^5^ Brigham and Women's Hospital Boston, MA United States

**Keywords:** type 2 diabetes, text messaging, mobile phones, physical activity, engagement, pedometers

## Abstract

**Background:**

Text messages are increasingly being used because of the low cost and the ubiquitous nature of mobile phones to engage patients in self-care behaviors. Self-care is particularly important in achieving treatment outcomes in type 2 diabetes mellitus (T2DM).

**Objective:**

This study examined the effect of personalized text messages on physical activity, as measured by a pedometer, and clinical outcomes in a diverse population of patients with T2DM.

**Methods:**

Text to Move (TTM) incorporates physical activity monitoring and coaching to provide automated and personalized text messages to help patients with T2DM achieve their physical activity goals. A total of 126 English- or Spanish-speaking patients with glycated hemoglobin A_1c_ (HbA_1c_) >7 were enrolled in-person to participate in the study for 6 months and were randomized into either the intervention arm that received the full complement of the intervention or a control arm that received only pedometers. The primary outcome was change in physical activity. We also assessed the effect of the intervention on HbA_1c_, weight, and participant engagement.

**Results:**

All participants (intervention: n=64; control: n=62) were included in the analyses. The intervention group had significantly higher monthly step counts in the third (risk ratio [RR] 4.89, 95% CI 1.20 to 19.92, *P*=.03) and fourth (RR 6.88, 95% CI 1.21 to 39.00, *P*=.03) months of the study compared to the control group. However, over the 6-month follow-up period, monthly step counts did not differ statistically by group (intervention group: 9092 steps; control group: 3722 steps; RR 2.44, 95% CI 0.68 to 8.74, *P*=.17). HbA_1c_ decreased by 0.07% (95% CI –0.47 to 0.34, *P*=.75) in the TTM group compared to the control group. Within groups, HbA_1c_ decreased significantly from baseline in the TTM group by –0.43% (95% CI –0.75 to –0.12, *P*=.01), but nonsignificantly in the control group by –0.21% (95% CI –0.49 to 0.06, *P*=.13). Similar changes were observed for other secondary outcomes.

**Conclusion:**

Personalized text messaging can be used to improve outcomes in patients with T2DM by employing optimal patient engagement measures.

## Introduction

### Background

The prevalence of type 2 diabetes mellitus (T2DM) in adults in the United States has more than quadrupled from 5.5 million in 1980 to 21.3 million in 2012 with an estimated total cost of US $245 billion [1]. To achieve the treatment goal of preventing or delaying complications of chronic disease, diabetes requires extensive multiple behavioral adjustments and self-care behaviors [1-3]. Today, diabetes education programs are offered in a variety of settings to equip patients with the knowledge and skills needed to modify their behavior and successfully self-manage the disease. However, physical activity (PA) and nutritional changes are more difficult for patients because of barriers such as socioeconomic factors, inadequate knowledge, lack of insight and motivation to change, or frustrations about inability to maintain consistent change [2,4].

It is well established that regular PA is effective in facilitating the attainment of treatment goals in the management of T2DM [4-6]. PA is associated with reductions in low-density lipoprotein cholesterol, systolic blood pressure, weight, symptoms of depression, and risk of cardiovascular all-cause mortality, and is associated with improvement in health-related quality of life [5,6]. Unfortunately, patients with T2DM are less likely to engage in regular PA, with recent estimates demonstrating a lower participation rate compared to the national average [7]. Given the growing number of patients with T2DM who are obese or have low levels of PA, improvements in this single behavior could have significant impact on overall outcomes in diabetes management.

The American Diabetes Association recommends encouraging patients to partake in mild to moderate PA, and coaching may be most beneficial in helping patients adopt and maintain regular engagement in PA [5]. There is increasing evidence of the effectiveness of coaching to support and better engage patients in managing their health [8]. However, to achieve coaching objectives, the process requires frequent contact or communication between the coach and the patient, which may not be feasible in an already overburdened health care system. In this project, we leveraged two key connected health cornerstones—objective data collection and targeted feedback—to develop a PA coaching program. Studies have shown that compared with non-behavior change theory-based interventions, theory-based interventions tend to be more effective in changing behaviors because they can allow for tailoring of the intervention to the individual due to enhanced bidirectional engagement [9-11]. Therefore, we collected PA data by digital pedometers and delivered targeted feedback via text messages based on the individual’s PA data and the stage of change on the transtheoretical model of behavior change. We conducted a randomized clinical trial to test the hypothesis that T2DM patients assigned to a PA monitoring and text-messaging program will be more active and attain better clinical outcomes compared to a control group of patients not receiving text messages.

### Objectives

The primary objective of this trial was to evaluate the effectiveness of sending daily PA-focused text messages versus no text messages on PA, measured by pedometers, in patients with T2DM receiving care at 4 health care centers affiliated with a large academic medical center. Secondarily, we evaluated the effects of the intervention on glycated hemoglobin A_1c_ (HbA_1c_) levels, weight changes, PA behavior change, level of engagement in the program, and the patient’s perception of usability and satisfaction with the text-messaging program.

## Methods

### Study Oversight

The study was approved by the Partners HealthCare Human Research Committees, the Institutional Review Board (IRB) for the Massachusetts General Hospital. All participants provided written informed consent.

### Participants

Participants were recruited from 4 health centers affiliated with a large academic medical center that serves a highly diverse population with high proportions of low-income and ethnic minorities. Eligible participants were English- or Spanish-speaking patients, aged 18 years and older, with a diagnosis of T2DM and most recent HbA_1c_ >7.0%. They had to have a computer with Internet access at home or at work, be willing to attend 2 in-person study visits, and also be willing to receive a minimum of 60 text messages per month for 6 months on their personal mobile phone. We excluded patients with significant cognitive deficits, physical disabilities, and medical or other surgical conditions precluding participation in moderate PA.

### Trial Design

The Text to Move (TTM) study was a 2 parallel group randomized controlled trial conducted from July 2012 to October 2013. The trial consisted of 2 study visits timed to coincide with a scheduled clinic appointment with their primary care providers (PCPs): screening/enrollment at the beginning of the study and a 6-month follow-up visit at the end of the study. All study materials, including the consent form, were translated into Spanish by an IRB-approved, certified Spanish translator. Participants received a check for US $50 at the end of each study visit.

### Screening and Enrollment

Primary care providers and diabetes self-management educators at the study sites were informed about the study and asked to refer potentially eligible patients for participation. A study staff member also reviewed TopCare, Partners HealthCare’s Web-based population registry for the management of patients with diabetes, to identify potential candidates. The list of potential participants identified from TopCare was sent to the managing PCPs for approval. All patients with T2DM, approved by their PCPs, were sent a recruitment letter with a 1-week opt-out option to inform the study team of their availability or nonavailability to participate in the study. Interested patients were prescreened by telephone for eligibility by research assistants using standardized scripts; eligible patients were invited for the in-person enrollment visit.

The enrollment visit lasted approximately 30 to 45 minutes and was conducted by research assistants in semiprivate rooms at each of the practices. Standardized enrollment procedures included rescreening to ascertain eligibility, informed consent procedures, on-the-spot HbA_1c_ self-check (Bayer HbA_1c_ Now), and completion of 3 study questionnaires:

1. Enrollment questionnaire: to collect baseline demographic information);

2. Physical activity Stages of Change Questionnaire: based on the transtheoretical model of change and assesses the motivational readiness of PA behavior change [12]; and

3. Patient Health Questionnaire (PHQ-8): a screener for depression [13].

Screening for third grade-level reading ability was done by testing the participant’s comprehension of sample study text messages. Also at this visit, participants received the study devices consisting of a study pedometer (ActiPed+) and accompanying Bluetooth wireless technology-enabled Universal Serial Bus (USB) connection device (ActiLink USB wireless stick) and device user guides. The study pedometer served only to capture or track activity data; it did not deliver any form of personalized feedback to participants.

The pedometer used in this study was the FitLinxx activity-tracking device, called the ActiPed+, which is available for consumer use. The ActiPed+ is a small, wireless activity sensor that clips onto any shoe and accurately tracks steps, distance traveled, calories burned, and activity time. The pedometer data were uploaded via the ActiLink USB wireless stick to the device Web portal [14] where participants could view their PA data on their personal account and modify their PA goals. Images of the devices and portal are included in Multimedia Appendices 1 and 2. The ActiPed+ has capacity to store up to 3 weeks’ worth of data. To view or download activity data from the pedometer, an ActiLink USB wireless stick needs to be installed on a computer with Internet access. The data automatically uploads any time the participant gets within a few feet of the ActiLink USB stick. Participants were instructed to upload their step data as regularly as possible, but no longer than 3 days so that they could view their data online and receive timely feedback on their activity levels through the study text messages. The study staff showed participants how to use the device and the website and also instructed them to set PA goals that they could modify on a monthly basis. However, the recommended PA goal of 30 minutes per day for at least 5 days in a week was preset for all participants [15].

### Randomization

After eligible patients signed the consent form, they were randomly assigned to receive the TTM intervention or to the control group with a 1:1 allocation ratio. A computer-generated permutated block randomization schedule, with block sizes ranging from 2 to 10, was established with STATA 12′s ralloc procedure. A third party, not involved with the study, randomly picked blocks and treatment assignments then concealed them in numbered opaque envelopes. Thus, study staff were not aware of treatment assignment before the participant opened the opaque randomization envelope at the enrollment visit. Similar to many technology-based studies, study participants and research assistants were not blinded to treatment assignments, but the investigators were not aware of treatment assignments.

The intervention (TTM) group participants received the study text messages with activity feedback, a study pedometer (plus connection device) to monitor their daily activity, reminder telephone calls to those participants who do not upload their activity data after 5 consecutive days, and usual care. Participants assigned to the control group received a study pedometer (plus connection device), reminder telephone calls for those participants who did not upload their activity data after 5 consecutive days, and usual care, but did not receive the study text messages with activity feedback.

### Follow-Up

Follow-up visits were conducted in-person by research assistants at the end of the 6-month study period. At this visit, participants completed the study surveys, had their follow-up HbA_1c_ test, and returned all study equipment. The follow-up questionnaires consisted of the Physical Activity Stages of Change Questionnaire and study-specific usability and satisfaction questionnaires.

### The Intervention

The intervention consisted of at least 2 automated text messages per day—one in the morning (weekdays: 9 am EST; weekends: 11 am EST) and a second message in the evenings at 6 pm EST. The messages were designed to provide bite-sized (160-character length) coaching based on daily step counts, captured by the pedometers, and preset PA goals which were agreed on at the initial visit. Additionally, at the initial visit, we collected baseline demographic and behavioral information that was entered into the text-messaging system to tailor the messages to participants. In all, a bank of more than 1000 text messages was designed by an interdisciplinary team of physicians, nurses, behavioral psychologists, health educators, health coaches, and social workers. The text messages were designed using health literacy concepts so they could be understood at a third grade reading level and were also available in Spanish. The Spanish translations went through a rigorous process to ensure simplicity and accuracy and were translated by IRB-approved Spanish translators and reviewed by a bilingual physician and health educators. All study data, including outgoing and incoming text messages, PA, goals, and stage of change, were displayed on the study dashboard, which was monitored weekly by study staff.

Morning messages provided feedback based on the previous day’s activity. For a participant with activity data in the previous 24 hours, an example of activity feedback message was “TTM study: as of 8:27 am, you were active for 45 mins yesterday which is 75% of your daily goal.” For participants without activity data in the past 24 hours, they received a reminder to upload their activity data. A sample reminder message was “TTM study: A quick reminder to upload your pedometer data. Need help? Call xxx-xxx-xxx.” Afternoon and evening messages focused more on coaching themes, such as support, health education, motivation, and reminders to engage in healthy behaviors.

The text messages were designed to be targeted to an individual’s stage of behavior change as determined by the transtheoretical model of behavior change. A behavioral psychologist used grounded theory techniques to group the messages into different stages of behavior change and themes. Major themes included health education, motivation/self-efficacy, support, health assessment, and basic pedometer messages. The PA stage of behavior change questionnaire [12] was used to determine baseline stage of behavior change at the enrollment visit. For example, patients identified as being in the contemplation stage received a different combination of educational, motivational, and activity-related messages than patients in the action stage. For example, a participant in the contemplation stage might receive the message “TTM Study: Take a minute to consider these questions, ‘What are some benefits of becoming more physically active? What are the benefits of staying the same?’” Another participant in the action stage would receive a different kind of message, such as “TTM study: How can you add steps to your regular activity? Can you take the stairs instead of an elevator?” In general, the text messages suggested additional ways to engage in PA, such as dancing, gardening, walking to lunch, walking the dog, parking farther from the worksite or mall entrance, etc.

Participants’ transition to another stage of the behavior change model was assessed monthly and was determined by attainment of activity goals captured by pedometers (participant had to meet PA goal for at least 20 days in a month to transition to another stage) and also by responses to items from the physical activity stage of change questionnaire that was delivered via text message. A study staff monitored and made the change on the study dashboard.

To optimize engagement, some of the messages were designed to be interactive, 2-way messages with short structured responses that were sent out twice a week (Tuesdays and Thursdays). Some of the interactive messages focused on satisfaction with the program, health status, knowledge of PA, food intake, and medication adherence. Sample 2-way messages included: “How would you rate your stress level over the last few weeks? 1=no stress 2=some stress 3=moderate stress 4=a lot of stress.” A response from the participant generated an automatic follow-up response from the system that completed the series of that interaction. For example, a participant who responded “3″ to the preceding question received the message: “Sounds like a lot to handle, how about talking with your doctor about stress management tools?”

### Outcome Assessments

The primary outcome for this study was mean step counts (collected by the wireless pedometers) per month for the entire 6-month study duration. Secondary outcomes included comparison of HbA_1c_ test results collected at enrollment and closeout visits. We also evaluated changes in weight (lb) measured at the clinic visit and collected from the medical records and PA stage of behavior change via the physical activity stage of change questionnaire [12]. In the intervention group, we also assessed usability and satisfaction by study-specific questionnaires and engagement with the intervention by the number of days that participants wore their pedometers in the study and the response rate to the 2-way interactive text messages. We further assessed engagement as a dichotomous outcome by classifying participants who responded to at least 1 text message per week for the entire 6-month duration as “engaged,” whereas those who did not respond to at least 1 message per week were regarded as “unengaged.”

### Sample Size

We calculated a sample size of 120 (60 participants per group) would be sufficient to detect a true difference of 1500 in mean step count between the control and intervention arms with 80% power and a 2-sided .05 significance level. This was based on the assumption that the standard deviation of the response variable was 2600 in both groups and was adjusted for a dropout rate of 20% [16]. Power calculations were performed in Stata 12 (StataCorp LP, College Station, TX, USA).

### Statistical Analysis

Only participants who completed closeout procedures were included in the final analyses. From initial testing, we observed that the pedometer registered some minimal steps (usually <100 steps) even when unused. Therefore, to differentiate real activity data (step counts) from “noise” data, we removed all step counts that were less than 100 steps. The intention-to-treat principle was used and participants were analyzed in the treatment group to which they were allocated. The last observation carried forward method was used for missing data from dropouts and loss to follow-up. Descriptive statistics, means (continuous data), and percentages (categorical variables) were used to summarize baseline characteristics by treatment group. Characteristics were compared between the 2 groups using independent *t* tests or chi-square tests as appropriate. The primary outcome, monthly step counts, was log transformed for normalization. Thereafter, we performed a repeated-measure procedure in SAS (PROC MIXED) for overall effect comparison between the 2 treatment groups, the monthly variation of step counts, and the interaction of group and time for the 6-month study duration. Least-square means of the log-transformed monthly step counts were back-log transformed to generate final estimates of least-square means. To control for baseline differences in HbA_1c_, an analysis of covariance, with follow-up HbA_1c_ at the end of the 6-month study period as the dependent variable and baseline HbA_1c_ and treatment group as independent variables, was performed [17]. Furthermore, we evaluated the response rate to the 2-way text messages among the intervention participants. We dichotomized the response rate to create 2 subgroups among the TTM group, engaged and unengaged participants, and examined the impact of text message response rate on daily activity and HbA_1c_ values. Data analyses were done with SAS version 9.3 (SAS Institute, Cary, NC, USA). All tests were 2-tailed and *P* values less than .05 were considered statistically significant.

## Results

### Participant Flow, Baseline Data, and Numbers Analyzed

**Figure 1:**
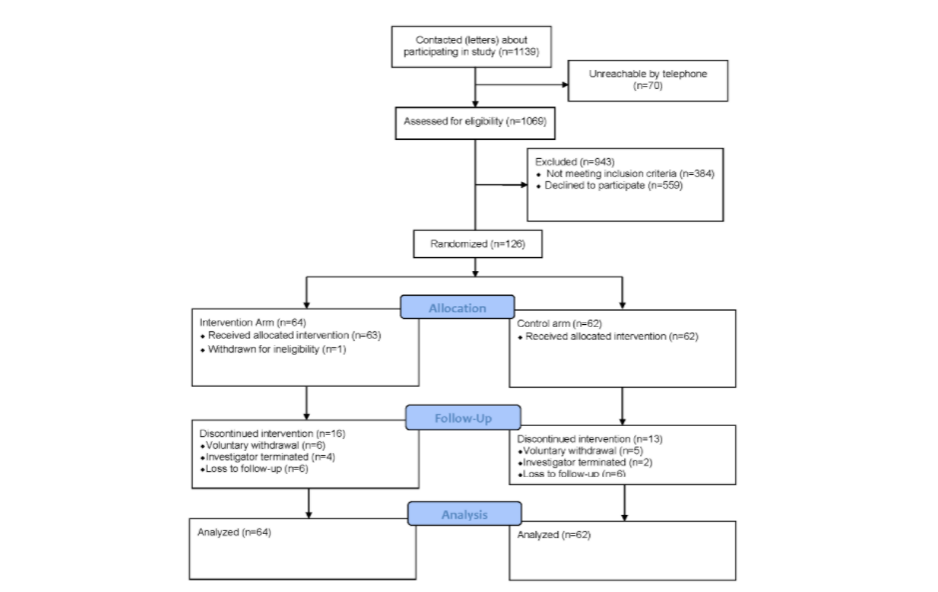
Participant flowchart.

Figure 1 is a flowchart describing the participant recruitment process. Between July 2012 and March 2013, a total of 1139 patients from the participating health centers that were approved by their PCPs were contacted about participating in the study. Of these, 70 patients were unreachable by telephone after recruitment letters were sent out to them, 559 patients were not interested in participating, 364 were ineligible at telephone prescreening with reasons ranging from no cell phone to physical limitation that precluded participation in moderate activity, and an additional 20 patients were found to be ineligible at the enrollment visit (primarily HbA_1c_ <7% and low health literacy).

A total of 126 participants were enrolled in the study and randomized to the control or intervention arm of the study. Of the total that enrolled, 12 participants withdrew voluntarily from the study. In the TTM group, reasons for withdrawal included hospitalization (n=1), loss of interest in continuing participation (n=2), pedometer-related problems (n=2), and loss of computer (n=2). In the control group, reasons for withdrawal included hospitalization (n=1), disappointment for not being assigned to the TTM group (n=1), memory loss (n=1), pedometer-related problem (n=1), and loss of interest (n=2). A participant who signed the consent form and was randomized to the TTM group was withdrawn from the study because she did not meet the HbA_1c_ eligibility criterion of >7%. This was discovered before the participant was enrolled in the text-messaging program. Six participants met prespecified drop criteria. Reasons for termination included inability to receive text messages on phone (n=1), inability to download the pedometer software (n=2), no longer had a computer (n=2), and no longer had Internet connection (n=1) and therefore had no means of uploading step counts. Participants who failed to attend the final study visit despite multiple contact attempts by study staff (n=12) were regarded as lost to follow-up. A total of 95 participants completed closeout procedures between February 2013 and October 2013. We analyzed data for all enrolled participants; their baseline characteristics are summarized by treatment arms in Table 1. The 2 groups were not statistically different at baseline.

**Table 1:** Baseline participant characteristics (N=126).

Characteristics		Intervention (n=64)	Control (n=62)	*P* value
Age (years), mean (SD)		50.3 (10.5)	52.6 (12.6)	.26
**Gender, n (%)**				.11
	Female	28 (44)	37 (60)	
	Male	36 (56)	25 (40)	
**Race, n (%)**				.56
	Asian/Pacific Islander	3 (5)	0 (0)	
	African-American	5 (8)	7 (11)	
	Hispanic	15 (23)	16 (26)	
	White	39 (61)	38 (61)	
	Other	2 (3)	1 (2)	
**Language, n (%)**				.23
	English	54 (84)	46 (74)	
	Spanish	10 (16)	16 (26)	
**Marital status, n (%)**				.88
	Divorced/Separated	12 (19)	10 (16)	
	Living with partner	7 (11)	5 (8)	
	Married	31 (48)	36 (58)	
	Single (never married)	11 (17)	9 (15)	
	Widowed	3 (5)	2 (3)	
**Education,^a^** **n (%)**				.06
	Grade 1-8	4 (6)	6 (10)		
	Grade 9-11	6 (9)	5 (8)	
	Grade 12 or GED	28 (44)	13 (22)	
	1-3 years of college	18 (28)	19 (32)	
	≥4 years of college	8 (13)	17 (28)	
**Employment, n (%)**				.24
	Employed full time	33 (52)	32 (52)	
	Employed part time	8 (13)	6 (10)	
	Unemployed	9 (14)	12 (19)	
	Homemaker	4 (6)	3 (5)	
	Retired	3 (5)	7 (11)	
	Disabled	4 (6)	0 (0)	
	Student	1 (2)	0 (0)	
	Other	2 (3)	2 (3)	
**Health center, n (%)**				.67
	Charlestown	8 (13)	10 (16)	
	Chelsea	21 (33)	25 (40)	
	Everett	14 (22)	10 (16)	
	Revere	21 (33)	17 (27)	
**PHQ-8 score,^a^** **n (%)**				.74
	0-4	46 (73)	41 (67)	
	5-9	13 (21)	15 (25)	
	10-14	1 (2)	3 (5)	
	15-19	2 (3)	2 (3)	
	20-24	1 (2)	0 (0)	
Weight (lb), mean (SD)		215.0 (56.8)	208.2 (46.9)	.53
**Enrollment season, n (%)**				>.99
	Winter	21 (33)	21 (34)	
	Spring	1 (2)	0 (0)	
	Summer	11 (17)	11 (18)	
	Fall	31 (48)	30 (48)	

^a^ Two participants in the control group had missing data.

### Outcomes and Estimation

Results showed that majority of the study population (67%, 84/126) had basal activity with mean daily step counts less than 2500 steps in the first week of the study. Over the 6-month follow-up period, the intervention group (9092 steps) had more overall monthly step counts than the control group (3722 steps), but this was not statistically significant (risk ratio [RR] 2.44, 95% CI 0.68 to 8.74, *P*=.17). Table 2 presents between-group differences of least-square means of the monthly step counts and Table 3 presents median monthly step counts. Within each group, monthly step counts decreased significantly from baseline to the end of the study: from 35,786 steps to 1041 steps in the intervention group and from 31,002 steps to 342 steps in the control group. Over the study period, monthly step counts varied between groups. In particular, we observed significant differences in the third and fourth month of the study. The intervention group had significantly higher monthly step counts in the third (RR 4.89, 95% CI 1.20 to 19.92, *P*=.03) and fourth (RR 6.88, 95% CI 1.21 to 39.00; *P*=.03) months compared to the control group.

**Table 2:** Total monthly least squares means of step counts.

Month	Intervention, least squares means	Control, least squares means	Effect estimate, RR (95% CI)	*P* value
1	35,786	31,002	1.15 (0.36 to 3.73)	.81
2	31,138	13,493	2.31 (0.59 to 9.08)	.23
3	37,436	7653	4.89 (1.20 to 19.92)	.03
4	14,254	2072	6.88 (1.21 to 39.00)	.03
5	913	1170	0.78 (0.10 to 6.37)	.82
6	1041	342	3.04 (0.36 to 25.93)	.31

**Table 3:** Median monthly step counts.

Month	Intervention, median (IQR)	Control, median (IQR)
1	85,509 (40,384-121,720)	60,967 (34,327-120,384)
2	59,467 (34,852-121,160)	52,117 (23,041-101,889)
3	73,927 (22,670-134,866)	36,610 (11,000-86,940)
4	46,003 (11,228-76,386)	22,738 (0-96,011)
5	8485 (0-66,550)	17,665 (0-75,823)
6	14,180 (0-74,302)	8220 (0-56,150)

Between groups, baseline mean HbA_1c_ (Table 4) was significantly higher in the TTM group (mean 9.02%, SD 1.63 vs mean 8.38%, SD 1.37; mean difference 0.64%, 95% CI –0.11 to 1.17, *P*=.02), but follow-up HbA_1c_ was not statistically different between groups (8.59%, SD 1.60 vs 8.17%, SD 1.60; difference: mean 0.42%, 95% CI –0.14 to 0.99, *P*=.14). After adjusting for baseline differences, HbA_1c_ decreased by 0.07% (95% CI –0.47 to 0.34, *P*=.75) in the TTM group compared with the control group. Within-group differences showed that HbA_1c_ decreased significantly from baseline in the TTM group by –0.43% (95% CI –0.75 to –0.12, *P*=.01) and nonsignificantly in the control group by –0.21% (95% CI –0.49 to 0.06, *P*=.13), but these pre-post changes were statistically different by group (mean difference 0.22%, 95% CI –0.19 to 0.64, *P*=.29). Follow-up weight was not significantly different by group (TTM: mean 211.99, SD 53.93 lb; control: mean 208.89, SD 48.59 lb; mean difference 3.10 lb, 95% CI –24.50 to 18.30, *P*=.77).

**Table 4:** Glycated hemoglobin A_1c_ (HbA_1c_).

Follow-up period		TTM (%), mean (SD)	Control (%), mean (SD)	Mean difference (95% CI)	*P* value
Baseline		9.02 (1.63)	8.38 (1.37)	0.64 (–0.11 to 1.17)	.02
Closeout		8.59 (1.60)	8.17 (1.60)	0.42 (–0.14, 0.99)	.14
	Change scores	–0.43	–0.21	0.22 (–0.19 to 0.64)	.29
	ANCOVA			–0.07 (–0.47 to 0.34)	.75

Table 5 shows the participants’ perception of their stage of behavior change. None of the participants identified as being in the precontemplation stage. At baseline, there were no significant differences by group. However, in the follow-up period, we observed that there was a greater proportion of TTM group participants in the contemplation stage compared with controls in that stage (25% vs 9.7%, *P*=.03).

**Table 5:** Stages of change on the transtheoretical model of behavior change.

Stages of change	Baseline			Follow-up		
	TTM, n (%)	Control, n (%)	*P*	TTM, n (%)	Control, n (%)	*P* value
Precontemplation	0 (0)	0 (0)		0 (0)	0 (0)	
Contemplation	23 (36)	21 (34)	.85	16 (25)	6 (10)	.03
Preparation	3 (5)	7 (11)	.20	8 (13)	10 (16)	.62
Action	4 (6)	2 (3)	.68	4 (6)	7 (11)	.36
Maintenance	34 (53)	32 (52)	>.99	36 (56)	39 (63)	.47

Engagement, as measured by number of days with pedometer data, did not differ by group. Overall, the TTM group wore their pedometers for a mean 109 (SD 40) days compared to a mean 97 (SD 56) days in the control group (mean difference 12, 95% CI 9.77-29.91, *P*=.32). Adherence to activity tracking measured by the proportion of participants with pedometer data (ie, participants wearing their pedometers) also varied by month (Table 6). It decreased from 93% (43/46) in the first month to 67% (31/46) at the end of the study in the TTM group; in the control group, this proportion decreased from 94% (46/49) in the first month to 55% (27/49) by the end of the study.

**Table 6:** Adherence to activity tracking: participants with activity data.

Month	Intervention (n=46), n (%)	Control (n=49), n (%)	*P* value
1	43 (93)	46 (94)	>.99
2	43 (93)	43 (88)	.49
3	44 (96)	41 (84)	.09
4	42 (91)	35 (71)	.02
5	30 (65)	33 (67)	.83
6	31 (67)	27 (55)	.22

### Ancillary Analyses

We found that 78% (36/46) of participants in the TTM group responded to at least 1 of the 2-way messages that were sent over the course of the study period. In all, 16 of the participants (35%) from the TTM group engaged with the intervention by responding to at least 1 text message per week for the entire 6-month duration, whereas 30 participants did not engage with the intervention by responding to at least 1 message per week. Adjusting for baseline characteristics, we found that engaged participants, on average, had 1122 more daily step counts (95% CI 84 to 2160, *P*=.04) and also had greater reductions in HbA_1c_ levels (mean difference –0.78%, 95% CI –1.64 to 0.09, *P*=.08) compared with the unengaged participants.

**Figure 2:**
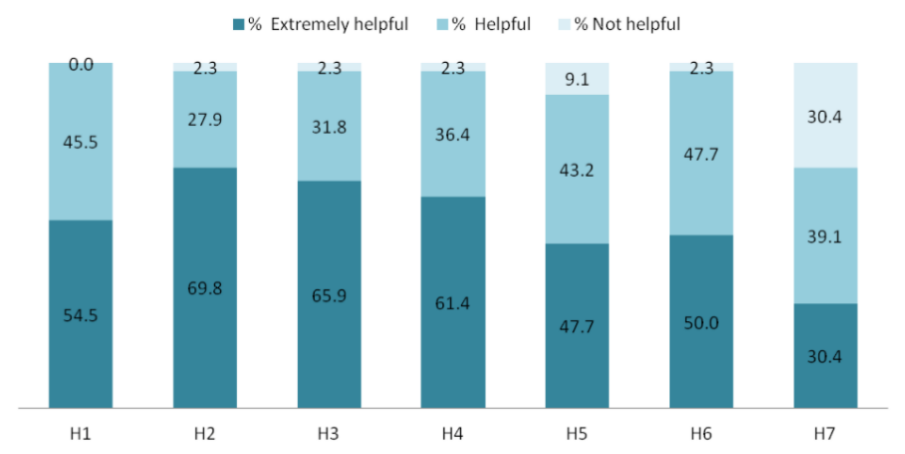
Participant perceptions of Text to Move. H1: providing educational information about PA; H2: giving feedback about number of step counts; H3: encouragement to increase level of PA; H4: reminders to be physically active; H5: asking questions that one could respond to; H6: helping one meet PA goals; H7: starting conversations about PA goals with doctor.

On a scale of 1 to 10, the overall mean participant rating of the usefulness of TTM was 8.62 (SD 1.79, range 4-10). A great majority of participants (94%, 43/46) would recommend TTM to their friends, 72% (33/46) reported that they would like to keep using the program, and 78% (36/46) would buy it for themselves or for another if it were for sale. The majority of participants who used the intervention found it helpful in improving their PA behaviors as shown in Figure 2.

Of the TTM users, 72% (33/46) of participants discussed their use of TTM with friends and family. They were generally well-supported by their social networks to use the intervention, with most participants receiving encouragement from friends and family (72%) and weekly reminders from them to engage in more PA (67%). Also, 63% (29/46) of participants discussed TTM with their PCPs.

More than half of participants (57%, 26/36) did not report any problems using TTM. Some of the problems experienced included problems with the USB connection device (n=7), difficulty uploading step counts (n=7), viewing step counts online (n=4), receiving text messages (n=2), and responding to text messages (n=4). For overall improvement of the text-messaging program, 26% (12/46) of participants enjoyed the program as it was and would not recommend any modifications. However, 17% (8/46) of participants wanted to see improvements in the text-messaging intervention. Specifically, they want the messages to be less repetitive and wanted to see more messages at different times of the day, such as additional messages at lunchtime. Additional recommendations included more opportunities to speak with a live person (9%, 4/46) and improved step count functionality (9%, 4/46). The remaining 33% (15/46) either did not respond or had no suggestions to improve the program.

## Discussion

Several industries are now able to leverage large amounts of data to provide intelligent and personalized information to consumers. This study attempted to use similar principles to personalize feedback to patients to improve their level of PA. Compared with similar studies [18-20], this study is innovative and stands out for several reasons. First, participants received at least two automated text messages per day for the entire 6 months: morning messages reported on the previous day’s activity goal attainment and the afternoon/evening message served to educate, motivate, or assess the participant’s health. Second, the texts included bidirectional interactive messages sent twice per week to foster participant engagement. Third, the monthly PA stage of change assessments increased the dynamism and relevance of the text messages. Fourth, we were able to demonstrate monthly variations in PA behaviors and engagement in this mobile-based study, which could inform future intervention design and implementation.

This study did not find significant overall effects of targeted text messaging on improving PA over the 6-month period. However, the TTM group did have significantly higher monthly step counts than the control group in the third and fourth months of the study, perhaps suggesting an optimal intervention period or an untoward effect resulting from the differential use of pedometer, by group, in the fourth month of the study. One of the reasons for not detecting changes between the groups might be linked to the design of the study. Giving pedometers to the control group may have blunted the effect of the intervention. There is some evidence that shows that simply providing people with activity trackers is correlated with improvements in PA levels by up to 13% [21]. This is consistent with the well-known Hawthorne effect in which individuals change their usual behavior in response to their awareness of being observed [22]. We provided pedometers to our control group to be able to objectively measure PA rather than self-reported data. For our other important secondary outcomes, we found that participation in the TTM program helped participants significantly lower their HbA_1c_ as well as weight from baseline. However, when compared to the change within the control group, the difference was not significant. This could possibly be explained by the increase in PA in the control group resulting from the use of a pedometer.

Other technology-based studies evaluating the effect of PA in the management of T2DM have demonstrated that such interventions are indeed effective [23]. Only 3 of 15 studies included in a review of such interventions were mobile phone-based and all demonstrated nonsignificant increases in PA [24-26]. Similarly, all 3 studies demonstrated significant decreases in HbA_1c_ from baseline. Similar to this study, all 3 studies were randomized trials, but the TTM approach is different because none of these included interactive 2-way messaging, automated daily PA-focused messages, or a theoretical framework in their design. Another PA monitoring and text-messaging study by Newton et al [27] conducted with type 1 diabetic patients did not increase PA. Unlike the TTM study, this study sent messages once a week, did not include 2-way messages, and did not personalize the messages. Connelly et al [23] concluded that applying methods/features to promote adherence to the intervention is associated with greater benefits. This is in consonance with our findings that engaged TTM participants responding to interactive study messages had significantly higher daily step counts and lower HbA_1c_ levels compared to those who did not.

Adherence to wearing pedometers was high and similar in both groups at the beginning of the study but decreased over the course of the study period. This suggests that pedometers alone may not sustain engagement in activity behaviors. By the fourth month of the study, the TTM group was significantly more adherent in the use of their activity trackers compared to the control group suggesting that this might be an optimal intervention period for the TTM intervention. The importance of adherence to the intervention cannot be overemphasized. Engaging in the program resulted in significantly improved outcomes compared to participants who did not engage. Even after adjusting for potential confounders (eg, age, race, gender, baseline activity), we found that the difference in outcomes was significant. Our intervention only offered motivation through targeted education and coaching messages. This seems to have worked for a subset of the cohort, helping them stay engaged with the program. Future efforts could incorporate other motivational techniques (eg, incentives, social support) to engage a higher number of participants and improve the overall outcomes in the intervention group.

Some of the decrease in engagement could be related to technical difficulties. By the end of our study, approximately 67% of intervention participants had pedometer data compared with 55% in controls. This drop in adherence over time is a common occurrence in technology-based studies. Faridi et al [24] reported that only 25% of intervention participants used their pedometers for at least 75% of study duration, whereas Newton et al [27] reported that 37% of intervention participants stopped wearing pedometers by the end of study period. Technical difficulties and forgetting to wear study pedometers were identified as major barriers to optimal adherence in other studies, and was true for our study participants as well.

Today, activity-tracking sensors have been greatly improved. They are now available in a variety of user-friendly forms that can be easily worn for most of the day: bracelets, wristbands, belt hooks, in mobile phones, smartwatches, and so on. Improvements in our big data analytic capabilities can now help us deliver dynamic and highly personalized interventions to patients in more sophisticated ways [28]. For instance, instead of just providing coaching, advanced analytic methodologies could help us determine the appropriate motivational technique to use with patients and help deliver completely different interventions to different patients. Some could get an intervention focused on enhancing social support in their day-to-day diabetes care, whereas others could be incentivized for positive behaviors. These advanced techniques hold great promise and can increase the proportion of patients who will engage with such programs long term. Other factors that may influence adherence include the frequency and timing of messages. Although more frequent messages could serve as a useful reminder, it could also potentially have a nagging or irritating effect. Also, sending messages at a “good” time when participants can practice or “catch up” on activity could be potentially helpful to participants.

### Limitations

This study has a number of limitations. Firstly, the requirement of a computer with Internet access to upload activity data coupled with problems installing the pedometer software introduced a number of operational challenges that increased the attrition rate in this study—approximately 24%. High attrition rates are common in these types of studies; therefore, we anticipated this a priori and augmented our sample size. More so, there is no difference in participants who dropped out of the study compared with those who completed follow-up, which rules out selection bias. Secondly, the differential rate of adherence to activity tracker use in the fourth month of the study, whereby the control group was less adherent to using the activity tracker, could have led to a misclassification of outcome data in the control group if they were indeed active but just did not use the activity tracker. Thirdly, we observed group differences in baseline HbA_1c_ that could potentially bias comparisons of follow-up changes, but we used a statistical approach to control for this baseline difference. Fourthly, we did not collect height to account for body mass. We believe that the TTM intervention, which encourages mild-moderate activity, can be used by anyone regardless of body mass index. Fifthly, we did not evaluate the effectiveness of the different types/themes of messages. As a result, we are not able to tell from this study which of the daily feedback, reminders, or educational-motivational messages was directly responsible for study effects, but we do know that participants that responded to the 2-way messages achieved better outcomes compared to those who did not respond regularly to study messages. Finally, due to the self-report nature of the stage of change questionnaire, participants may have overestimated their stage of change at baseline and some participants might have received messages that were not appropriate for their actual stage of behavior change at the beginning of the study.

### Generalizability

Participants were recruited from 4 health care centers affiliated with a large academic medical center that serves a highly diverse population of ethnic minorities and immigrants. The areas served by these health centers also have some of the highest poverty levels in the state of Massachusetts. Apart from referring their patients to participate in the study, the care providers had no other formal role to play in the study. As such, the program can be implemented in various clinical settings as well as nonclinical settings. The pedometer technology was a limiting factor that introduced a number of operational challenges in implementing the study. However, the TTM program is not tied to any particular activity tracker and can easily integrate with any activity-tracking technology that is appropriate for the population under consideration.

### Conclusion

Text-messaging interventions that deliver targeted coaching, can be deployed on any type of phone (mobile phone or ordinary feature phones), and are feasible to develop and deploy can be used to engage patients with T2DM. Patients find such programs acceptable and a majority of patients were very satisfied with the intervention. Significant improvements in clinical outcomes can be obtained if such programs are able to achieve meaningful engagement in participants. The relatively low cost and ease of use makes it possible for such programs to be easily scaled and sustained for a longer duration across a diverse patient population regardless of age, educational, economic, or ethnic background. Future studies evaluating the effect of other personalization strategies, such as timing, optimal intervention period, frequency, and content of messages, will further help to improve adherence to such interventions. Also, strategies to use other motivational techniques could be explored to engage a larger subset of patients. Finally, efforts to integrate such care models into the workflow and usual care delivery of providers could be evaluated to help scale such programs in the future.

## Appendix

Multimedia Appendix 1 Pedometer image.

Multimedia Appendix 2 Actihealth portal.

## Abbreviations

IRB: Institutional Review Board
PA: physical activity
PCP: primary care provider
PHQ-8: Patient Health Questionnaire
RR: risk ratio
T2DM: type 2 diabetes mellitus
TTM: Text to Move
USB: Universal Serial Bus
