# Supplementary figures and images for: Text to Move: A Randomized Controlled Trial of a Text-Messaging Program to Improve Physical Activity Behaviors in Patients With Type 2 Diabetes Mellitus
Source: J Med Internet Res. 2016 Nov 18;18(11):e307. doi: 10.2196/jmir.6439 (PMC5135731; doi:10.2196/jmir.6439)

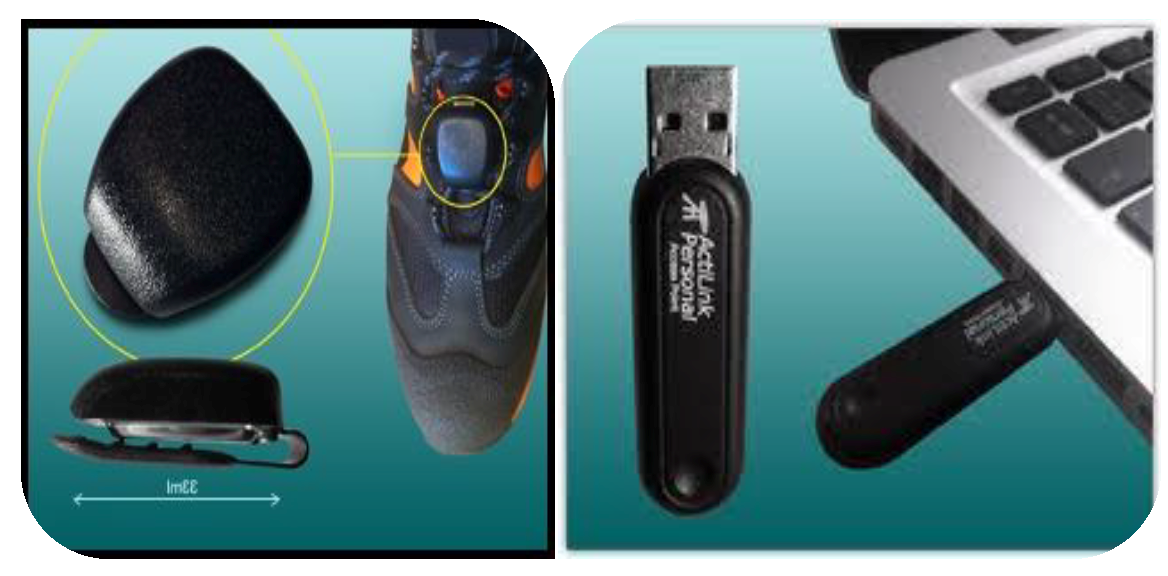

Supplement: Multimedia Appendix 1 [file jmir_v18i11e307_app1.png]

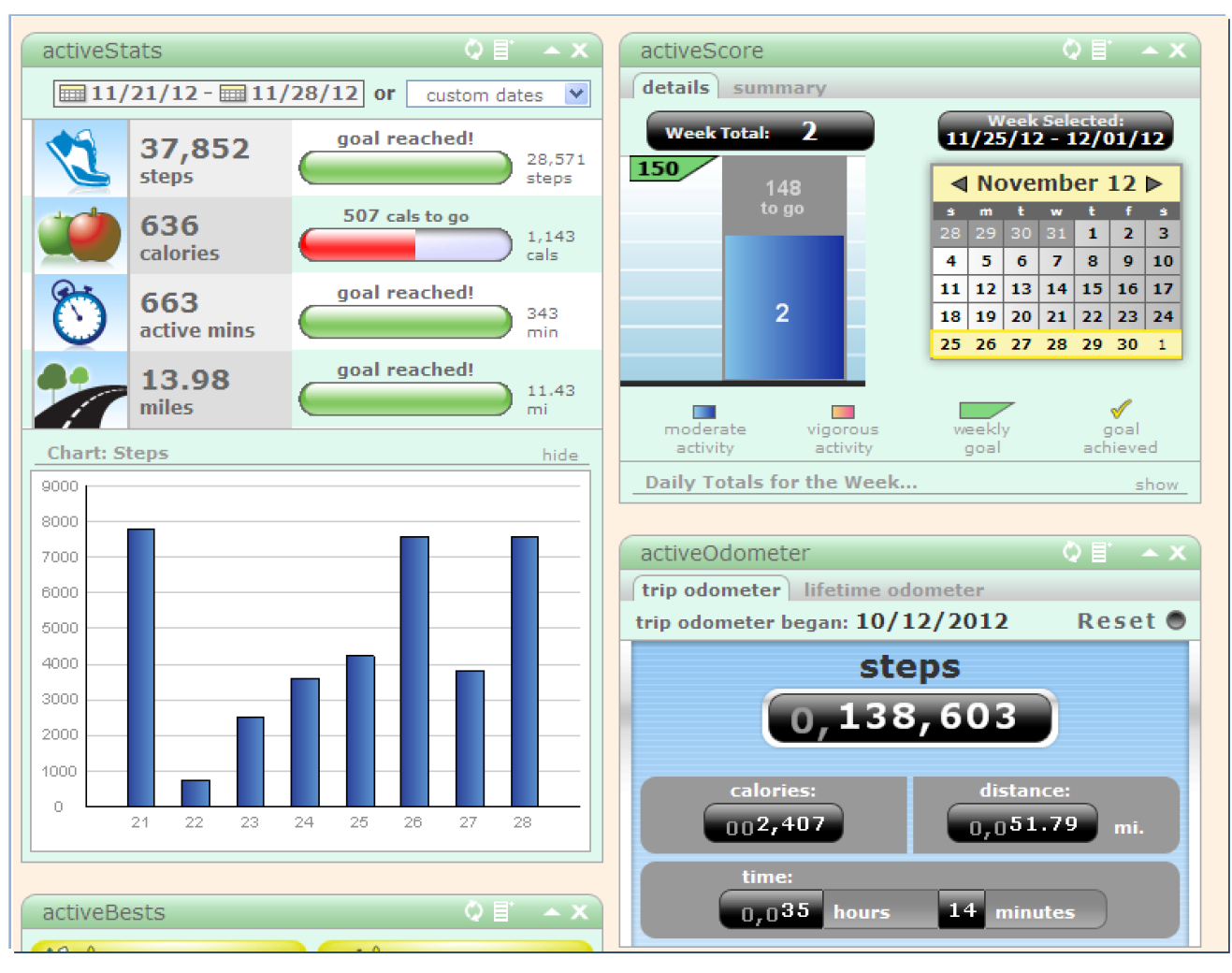

Supplement: Multimedia Appendix 2 [file jmir_v18i11e307_app2.png]
